# A Novel Lateral Flow Assay for Rapid and Sensitive Nucleic Acid Detection of *Avibacterium paragallinarum*

**DOI:** 10.3389/fvets.2021.738558

**Published:** 2021-10-11

**Authors:** Caiyun Huo, Donghai Li, Zhenguo Hu, Guiping Li, Yanxin Hu, Huiling Sun

**Affiliations:** ^1^Beijing Key Laboratory for Prevention and Control of Infectious Diseases in Livestock and Poultry, Institute of Animal Husbandry and Veterinary Medicine, Beijing Academy of Agriculture and Forestry Sciences, Beijing, China; ^2^Key Laboratory of Animal Epidemiology of Ministry of Agriculture, College of Veterinary Medicine, China Agricultural University, Beijing, China

**Keywords:** *Avibacterium paragallinarum*, lateral flow assay, nucleic acid detection, rapid, sensitive

## Abstract

*Avibacterium paragallinarum*, the pathogen of infectious coryza, caused a highly contagious respiratory disease that poses a serious threat to chickens. Hence, it is necessary to do diagnostic screening for *Av. paragallinarum*. Existing technologies have been used for *Av. paragallinarum* testing, which, however, have some drawbacks such as time consuming and expensive that require well-trained personnel and sophisticated infrastructure, especially when they are limitedly feasible in some places for lack of resources. Nucleic acid hybridization-based lateral flow assay (LFA) is capable of dealing with these drawbacks, which is attributed to the advantages, such low cost, rapid, and simple. However, nucleic acid determination of *Av. paragallinarum* through LFA method has not been reported so far. In this study, we developed a novel LFA method that employed gold nanoparticle probes to detect amplified *Av. paragallinarum* dsDNA. Compared with agarose gel electrophoresis, this LFA strip was inexpensive, simple- to- use, and time- saving, which displayed the visual results within 5–8 min. This LFA strip had higher sensitivity that achieved the detection limit of 10^1^ CFU/ml compared with 10^2^ CFU/ml in agarose gel electrophoresis. Besides, great sensitivity was also shown in the LFA strip, and no cross reaction existed for other bacteria. Furthermore, *Av. paragallinarum* in clinical chickens with infectious coryza were perfectly detected by our established LFA strip. Our study is the first to develop the LFA integrated with amplification and sample preparation techniques for better nucleic acid detection of *Av. paragallinarum*, which holds great potential for rapid, accurate, and on-site determination methods for early diagnosis of *Av. paragallinarum* to control further spreading.

## Introduction

Infectious coryza, caused by Gram-negative *Avibacterium paragallinarum*, is a severe respiratory disease of chickens that engenders huge economic losses for the poultry industry over the past decade (1, 2). Based on two kinds of schemes—the Page scheme and the Kume scheme, *Av. paragallinarum* can be serotyped into three serogroups (A, B, and C) and nine serovars (A1–A4, B-1, and C1–C4), respectively (3, 4). Besides, a previous study has also shown that great cross-protection exists among four serovars within serogroup A. However, cross-protection is weakened between some serovars within serogroup C (5, 6). In recent years, *Av. paragallinarum* has appeared in multiple countries, like the United States, Indonesia, India, and the United Kingdom. In China, all three serotypes of *Av. paragallinarum* have also been reported (6–8). The most common clinical features of the disease are sneezing, swelling of infraorbital sinuses, facial edema and conjunctivitis, nasal exudates, accompanied by growth retardation, and reduced egg production, which are tightly related to economic losses for the poultry industry. Thus, it is necessary for the development of reliable tools for *Av. paragallinarum* detection, which can facilitate diagnostic intervention in the early stage of infection and hereby reduce annual economic losses.

Up to now, various detection methods have been used for the detection of *Av. paragallinarum*, including serological tests such as enzyme-linked immunosorbent assay (ELISA), nucleic acid tests, such as classic polymerase chain reaction (PCR) and quantitative real-time PCR (qRT-PCR). Unfortunately, several technical difficulties and challenges hinder the widespread use of these methods. For example, although ELISA is well-known as a high-throughput technique for bacteria detection, it has some drawbacks including requirement of highly specific and sensitive antibodies, time- consuming, and tedious washing and incubation steps (9). Thus, such ELISA method cannot be considered as an efficient method to be widely applied for *Av. paragallinarum*.

Compared with the conventional serological tests, nucleic acid detection technology is demonstrated to have the characteristics of high sensitivity and strong specificity, which has considerable advantages in shortening the detection time and improving the detection rate of pathogens (10, 11). Thus, it is an important supplement to the immunological detection methods. At present, the common nucleic acid detection technology is PCR, in which the amplification products obtained by this technology are initially determined by agarose gel electrophoresis based on the size of a specific band. However, some bottlenecks inhibit further application of the PCR method. For instance, traditional PCR method is a time-consuming process, tedious, and easy to produce pollution (12, 13). Notably, it seems that this method could hardly meet the requirements of on-site field detection. With the continuous development of molecular biology technology, various PCR-based technologies are developed, especially the invention of qRT-PCR, which has brought new vitality in the whole molecular diagnosis industry (14). This technology has the characteristics of high sensitivity and specificity, while requiring sophisticated infrastructure and experienced operator. To date, many fluorescence quantitative PCR detection kits on the market are designated with specialized equipment, which is expensive and has a single application. At the same time, related kit operators are required to have professional experimental skills and knowledge background, so this method is difficult to popularize. Subsequently, development of a novel *Av. paragallinarum*-specific, sensitive, rapid, low-cost, and high-throughput nucleic acid detection technology for early diagnosis of *Av. paragallinarum* is extremely meaningful for the control of further spreading.

Nowadays, lateral flow assay (LFA), one rapid and sensitive detection method, has become popular and extensively applied in various fields, which attributes to the advantages of low- cost, rapid, sensitive analysis, and simple with no need of sophisticated infrastructure and experienced operator (15). As a new type of immune labeling technology, the nanogold particle is commonly used as a trace marker to be applied to antigen and antibody in this technology (16). Nanogold is negatively charged in an alkalescence environment and can firmly bind with the positively charged groups of protein molecules, which does not affect the biological properties of proteins since the electrostatic binding. Except for protein binding, nanogold is also capable of binding to other macromolecules, such as phytohemagglutinin and concanavalin. Considering the physical characteristics of nanogold, like particle size, shape, and color reaction, coupled with the biological properties of the binder, it is extensively used in immunology, histology, pathology, cell biology, and other fields. By the design of LFA strips in the sandwich format, the LFA method has been very helpful for the detection of viruses, bacteria, and parasite antigens, smaller molecular drugs, and so on (17–20). For instance, it can be adopted to evaluate the toxins in different samples without tedious operation (21). In terms of avian pathogens, this method is capable of detecting influenza virus, Newcastle disease virus, avian leukosis virus, and so on (22–26). Nevertheless, most of these examples are mainly antibody tests or small molecule tests, and thus, LFA-based nucleic acid tests need to be further developed and improved.

More researchers have attempted to find a solution to make the advantages of LFA-based nanogold chromatography be applied to nucleic acid detection, such as the time-saving and simple operation (27, 28). In 1996, *Mirkin* et al. from Northwestern University in the United States prepared a gold–DNA complex probe according to the property of stable Au–S bond formed between gold nanoparticles and sulfhydryl, which can be used for DNA detection (29). Moreover, *Brittany A. Rohrman* et al. have successfully detected HIV nucleic acid amplification products using nanogold immunochromatography (30). They have designed three probes, one kind of probe is labeled with colloid gold particles that are regarded as a specific probe for hybridization with HIV-specific amplification product, the other two kinds of nucleic acid probes are coated on the test line and quality control line that are used to capture the HIV nucleic acid amplification products. In terms of bacteria, this method is also widely applied to detect the nucleic acid of the bacteria. For example, an LFA strip for the detection of avian pathogenic *Escherichia coli* serotype O78 (APEC O78) is established by *Wenfang Nie* et al., which can realize the rapid and on-site detection without requiring a sophisticated device and a well-trained operator (31). In terms of *Av. paragallinarum*, although a recent study has established an LFA strip to determine the bacteria in chickens with suspected infectious coryza, it belonged to a test for TonB-dependent transporter (TBDT) in bacteria cultures and clinical field samples, which is a common immunochromatography assay for small molecule detection (32). Here, to the best of our knowledge, nucleic acid determination of *Av. paragallinarum* through LFA method has not been reported so far.

Herein, we designed a novel amplification-integrated LFA strip for rapid and accurate determination of nucleic acid of *Av. paragallinarum*. Nanogold particles conjugated to mouse anti-digoxigenin antibody were used as probes. The sensitivity and specificity of LFA were assessed and compared with traditional agarose gel electrophoresis. The reliability and accuracy of LFA for nucleic acid detection of *Av. paragallinarum* in field-collected chickens with suspected infectious coryza was also evaluated with this new method. The results manifest that this LFA might be applied in conjunction with amplification for nucleic acid detection of *Av. paragallinarum* at clinically meaningful levels.

## Materials and Methods

### Bacterial Strains

Bacterial strains were demonstrated as follows: *Av. paragallinarum, Ornithobacterium rhinotracheale, Enterococcus faecium, Escherichia coli, Avian streptococcosis, Brodetella bronchiseptica, Pasteurellamultocida, Mycoplasma gallisepticum, Av. gallinarum, Av. Avium*, and *Av. volantium*. They were all saved in the lab of Beijing Academy of Agriculture and Forestry Sciences.

### Pre-amplification of Genomic DNA

The strain 221 of the *Av. paragallinarum* serogroup A was cultured as previously described (1). First of all, the colonies of bacterial strains were resuspended in sterile water at 30 μl and then were boiled for 10 min. The bacteria at the concentration of gradient dilution were directly boiled for 10 min, and then placed on ice for 10 min. The supernatant could be used as genomic DNA (33). For amplification, target template was amplified with the specific biotin-digoxin labeled primer set. The sequences of this primer set were as follows: N1-B1 (forward primer, 5′-B1-TGAGGGTAGTCTTGCACGCGAAT-3′); R1-D1 (reverse primer, 5′-DI-CAAGGTATCGATCGTCTCTCTACT−3′). Typically, each reaction contained 12.5 μl 2 × Taq PCR Mix with loading dye, 0.5 μl 10 μM of forward primer, 0.5 μl 10 μM of reverse primer, 1 μl template, and 10.5 μl sterile water. The thermal cycling conditions were as follows: 94°C 5 min; 94°C 30 s, 56°C 30 s, 72°C 2 min (35 cycle); 72°C 10 min. The 10 μl final amplification products were validated by agarose gel electrophoresis.

### Preparation of Nanogold Particles

Nanogold particles were produced by trisodium citrate method as reported previously (21, 34). Briefly, 100 ml of 0.01% chloroauric acid solution (HAuCl_4_) was heated until boiling, and was then added with 0.75, 1, 1.5, 2, and 4 ml of 1% trisodium citrate solution, respectively. After it boiled for around 5 min, the color of the solution was changed from yellow to wine-red. Then, the solution was cooled and filtered. Here, the size of nanogold particle was identified by transmission electron microscopy (TEM) (15). Finally, the nanogold solution was stored at room temperature and 4°C, respectively. Notably, if there are floating objects on the surface of the solution, it indicates instability and poor degree of the solution and needs to be discarded.

### Preparation of Nanogold–Antibody Conjugates

The 3 ml nanogold solution was adjusted to optimal pH with 30 μl 2% potassium carbonate (21). The purified mouse anti-digoxigenin antibody (Roche, Germany) was diluted with phosphate buffer to 0.5 mg/ml and then was slowly added to the 3 ml nanogold solution for labeling with the concentration of 10 μg/ml. After reaction for 20 min, 15 μl of 20% BSA blocking solution was added with mild stirring for 10 min to stabilize the conjugate. The solution was centrifuged at 9,000 rpm for 30 min and resuspended to the 1.5 ml with conjugate dilution buffer (1% BSA and 0.02 M phosphate buffer) and stored at 4°C.

### Fabrication of Lateral Flow Assay Test Strip

LFA test strip was composed of three pads (sample, conjugate, and absorbent pads) and a nitrocellulose (NC) membrane with test and control areas. The following was the construction of the device: The goat anti-biotin antibody and goat anti-mouse IgG antibody (Sigma, America) were embedded onto the test (T) area and control (C) area of the NC membrane using the 0.01 M phosphate buffer (pH 7.4), respectively. The treated NC membrane was dried for 10 min at room temperature. Then, the sample pad, conjugate pad, NC membrane, and absorption pad were assembled into a laminated sheet sequentially with a 1- to 2-mm overlap. Finally, the sheet was cut into 4-mm wide strips. The strips were sealed in aluminum foil and stored at 4°C.

### Sensitivity and Specificity Analysis of the Test Strip

Before assessing the sensitivity of the test strips, the bacteria of *Av. paragallinarum* was quantified by the counting method. First, 100 μl of bacteria was diluted by serial 10-fold dilutions. Then, 100 μl of these diluted concentrations was added to the inoculated in the tryptic soy broth agar plates that were added with 10% chicken serum and 0.0025% NAD, respectively. Subsequently, the CFU/ml was determined by counting the numbers of bacteria. Based on the CFU/ml of bacterial, we could then dilute the bacteria from 10^4^ to 10^0^ CFU/ml with phosphate buffer solution, and then 20 μl pre-amplified products were mixed with 100 μl of running buffer and then dropped onto the sample pad. The running buffer was phosphate buffer solution at pH 7.4 that consisted of Na_2_HPO_4_, NaH_2_PO_4_, NACI, and water. After incubation for 5–8 min at room temperature, the results could be observed visually by the naked eyes. To evaluate the specificity of the test strips, pre-amplified products of all selected bacteria strains as mentioned above were used in the study using the same experimental procedures.

### Rapid and Accurate Determination of Clinical Sample by the Test Strip

For clinical samples, the swabs from the infraorbital sinuses of the chickens with suspected infectious coryza were collected. They were inoculated in the tryptic soy broth agar plates that were added with 10% chicken serum and 0.0025% NAD as previously described (1). The plates were incubated and suspected colonies of *Av. paragallinarum* were pre-amplified and detected using the same methods just as above mentioned. The handling of the animals followed the Guidelines of the Animal Care and Use Committee of Animal Husbandry and Veterinary Medicine of the Beijing Academy of Agriculture and Forestry Sciences (IAHVM-BAAFS).

## Results and Discussion

In this work, nucleic acid detection of *Av. paragallinarum* is the test object An LFA-based strip has been developed with the advantages of rapid, facile identification, cost-effectiveness, great portability, convenient visual judgment by the naked eyes as well as on-site determination.

### Mechanism and Establishment of Lateral Flow Assay Strip for Nucleic Acid Detection of *Av. paragallinarum*

**Figure 1:**
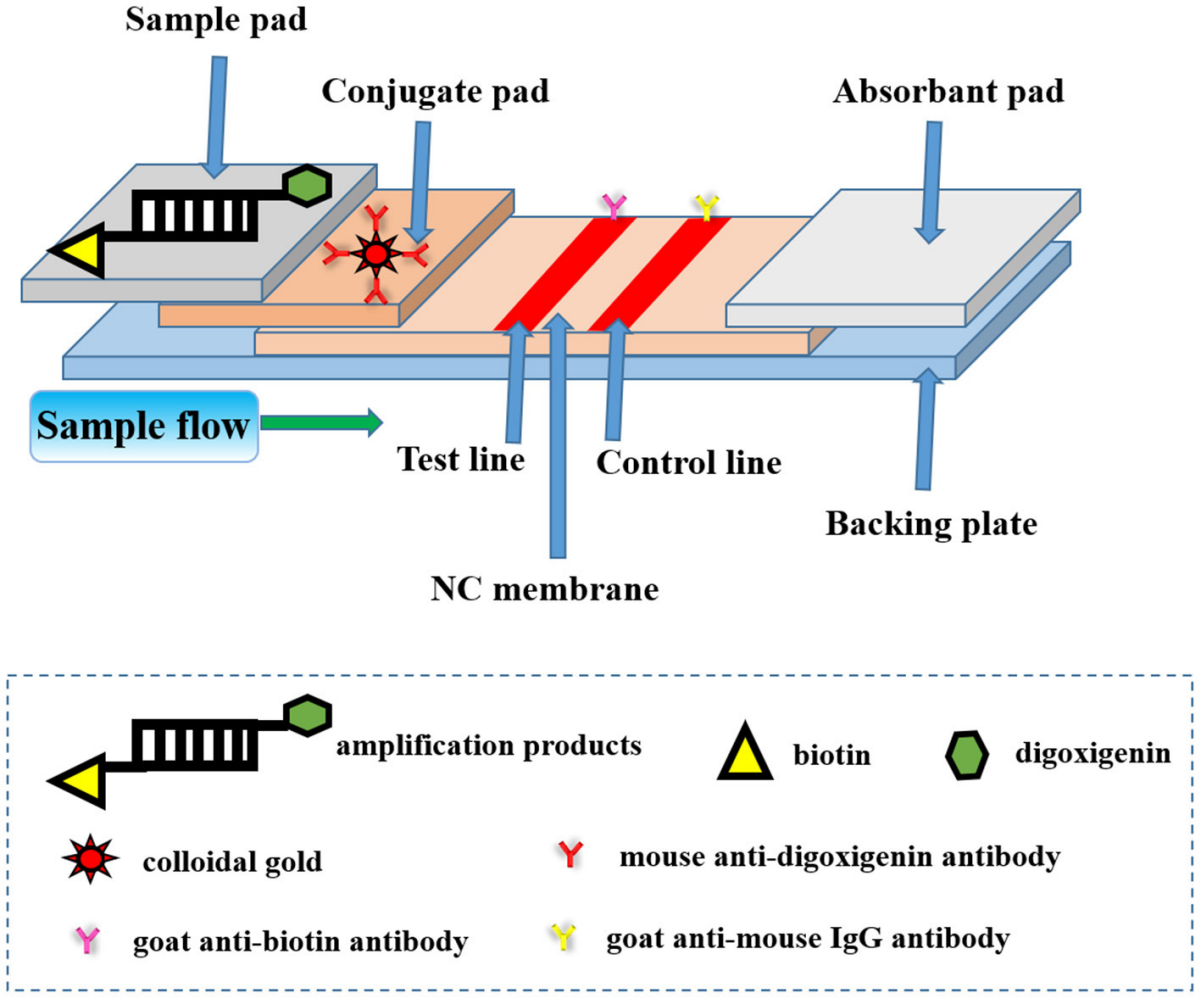
The schematic diagram of lateral flow assay (LFA) strip for nucleic acid detection of *Avibacterium paragallinarum*. The test strip included three pads (sample pad, conjugate pad, and absorption pad), an NC membrane, and a backing plate. The conjugate pad contained the nanogold-labeled mouse anti-digoxigenin antibody. The goat anti-biotin antibody and goat anti-mouse IgG antibody were embedded onto the test line and control line of the NC membrane, respectively. The dual-labeled dsDNA product of the bacteria could be added to the sample pad during the process of detection.

The principle of this LFA can be illustrated in Figure 1. The absorbent pad provided a lateral flow driving force according to capillary action and the test pad served as a platform for both bio-analytical reactions and identifications (15). Immobilized capture molecules like antibodies could be deposited on the surface of NC membrane to form a test line and a control line, respectively. Normally, conjugated pad was filled with labeling agents such as functional nanogold particles. Each two adjacent pads were laid to overlap each other to facilitate a continuous lateral flow immediately after the dual-labeled dsDNA product was added to the sample pad.

**Figure 2:**
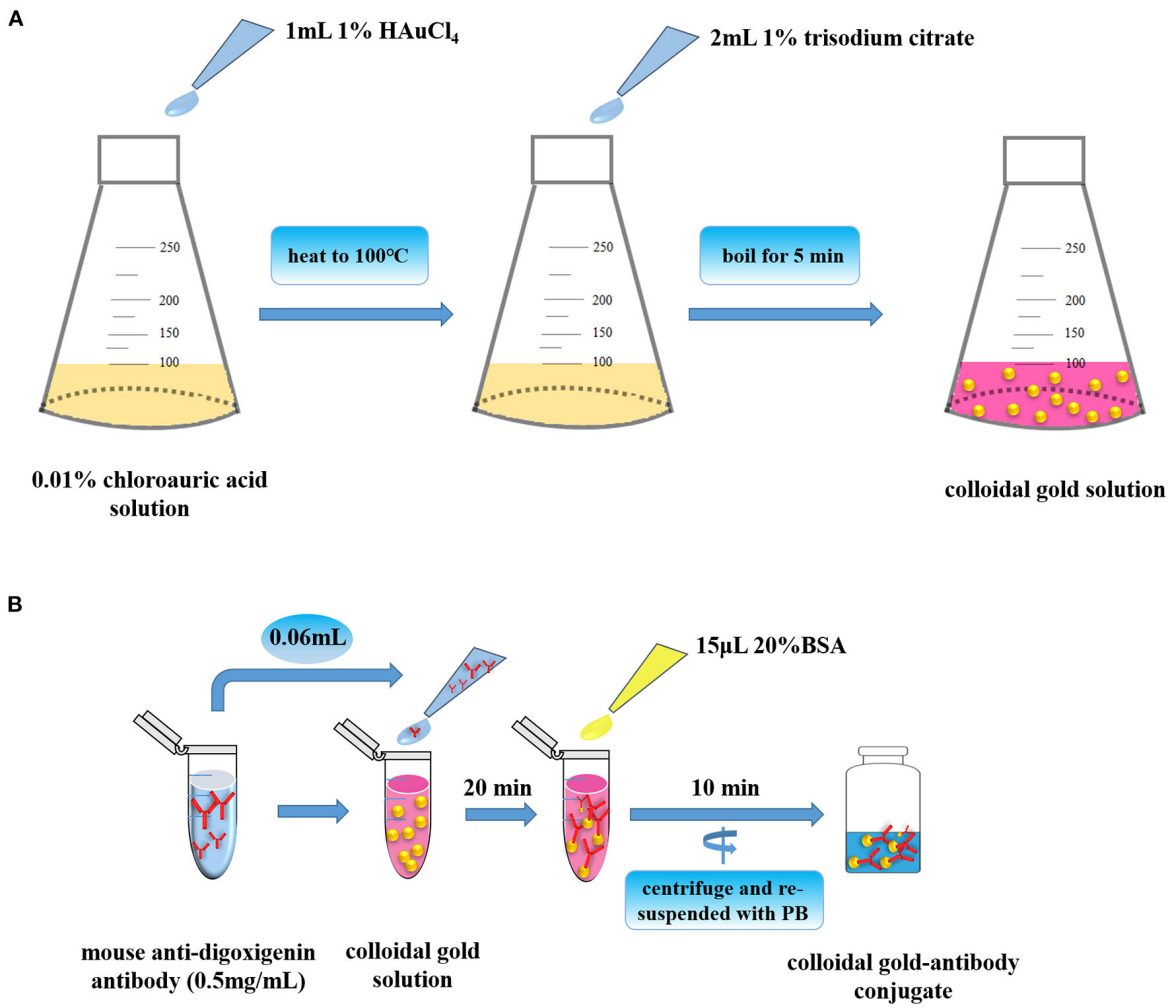
Preparation of nanogold and nanogold–antibody conjugates. **(A)** Preparation of high-quality colloid gold solution. **(B)** Preparation of high-quality colloid gold-antibody conjugates.

In the process of the establishment of LFA strip, the nanogold solution was first prepared as shown in Figure 2A. In the LFA strip, preparation of high-quality colloid gold solution is extremely essential for assurance of the sensitivity of the strip. In our study, the particles were about 25 nm in diameter, which provided a good basis for preparing the nanogold–antibody conjugate. Notably, the color of nanogold solution mainly relied on the volume of trisodium citrate used in its preparation process (15). It could be clearly seen by visual observation that the color of the nanogold solution became lighter from lavender to red with the increased addition of trisodium citrate, but there was no significant change in red color when the added amount was 2 and 4 ml. Thus, 2 ml of trisodium citrate could be selected as the optimal volume for its cost effectiveness and visualization performance eventually during the preparation of the nanogold solution in the following experiments. For storage condition, we compared the influence of storage temperature on the stability of this solution, and found that no floating objects were present on the surface of solution whether it was stored at room temperature or 4°C, indicating the great stability and degree of the nanogold solution. Subsequently, the nanogold–antibody conjugates were successfully prepared (Figure 2B). After a series of attempts, the optimum concentration of nanogold-labeled mouse anti-digoxigenin antibody was validated to be 10 μg/ml that could be finally selected for better labeling. Thereafter, the LFA strip was established by the method described above (31). In our LFA test strip, the goat anti-biotin antibody and goat anti-mouse IgG antibody were applied with the appropriate concentration for suitable labeling at 1 and 1 mg/ml, respectively. Therefore, the optimal conditions mentioned above were used in the following experiments. Finally, the LFA test strip for nucleic acid detection of *Av. paragallinarum* was assembled after all aforementioned parameters were optimized or determined.

**Figure 3:**
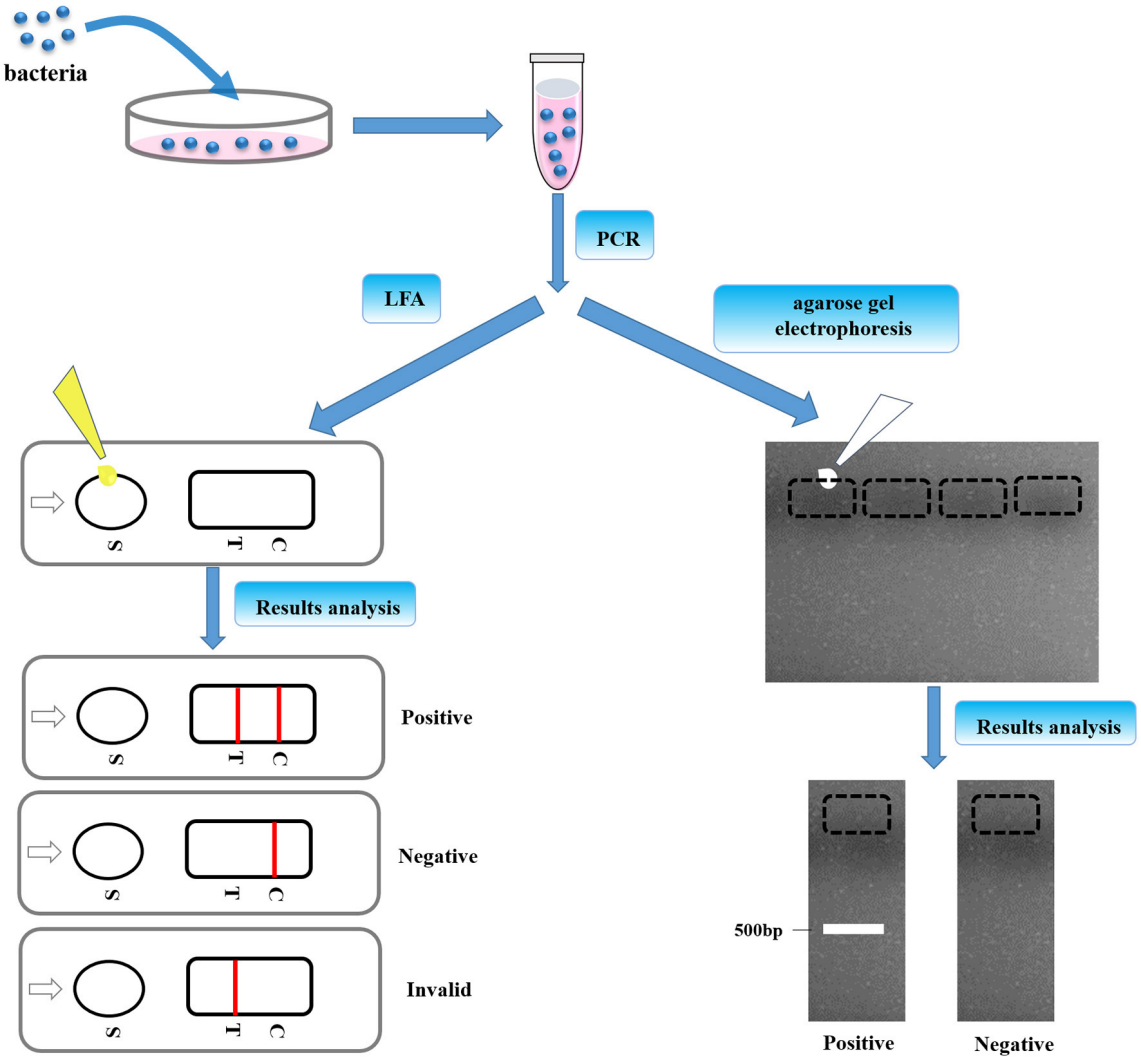
The procedures of LFA strip. The bacteria were cultured and pre-amplification of genomic DNA was taken with the specific biotin–digoxin-labeled primer set. Then, the final amplification products were dropped to the sample pad, which was also confirmed by agarose gel electrophoresis.

After that all aforementioned parameters were well-confirmed, the utility of this LFA strip was determined subsequently. The procedure of LFA strip for nucleic acid detection of *Av. paragallinarum* is schemed in Figure 3. After collection of bacteria and pre-amplification of genomic DNA with the specific biotin–digoxin-labeled primer set, the dsDNA products were capable of being labeled with the specific tags including biotin and digoxin at the same time. Subsequently, the final amplification products were then added onto the sample pad. If the bacteria in the sample are the *Av. paragallinarum*, this dual-labeled dsDNA product can be captured onto the test line by the pre-immobilized anti-biotin antibody and also further labeled with the nanogold–antibody conjugates on the basis of the immunorecognition principle. Thus, if the red color is shown on the test line, the sample is considered as the *Av. paragallinarum* positive. In contrast, if there is no *Av. paragallinarum* in the sample, the sample is regarded as the *Av. paragallinarum* negative. Due to that, the control area is utilized to validate whether the assay is accomplished correctly or not, so this area is generally red under the condition of precise operation even if target amplicons are not present in the detected samples. The results of the strip can be assessed within 5–8 min by the naked eyes without requirement of special equipment and trained operator. In spite of the test line showing red, this strip is regarded as invalid if no red line is seen in the control area. With this developed LFA, target nucleic acid of *Av. paragallinarum* could be well-determined. At the same time, traditional agarose gel electrophoresis was also used to verify the final amplification products to further validate the feasibility of the established LFA method.

### Sensitivity Analysis of Lateral Flow Assay Strip for Nucleic Acid Detection of *Av. paragallinarum*

**Figure 4:**
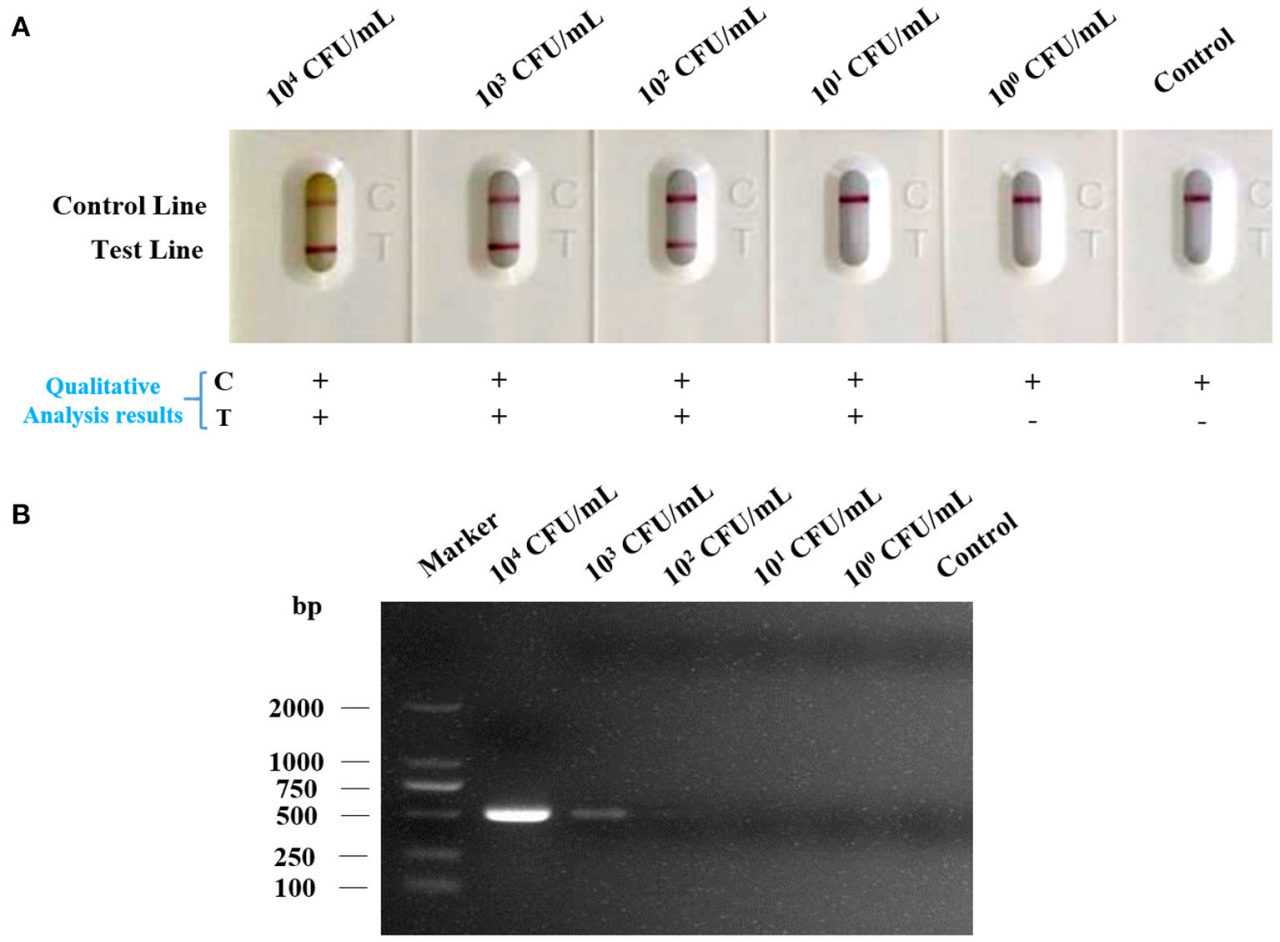
The sensitivity analysis of LFA strip. The cultured *Av. paragallinarum* was diluted from 10^4^ to 10^0^ CFU/ml with PBS, and then were detected by **(A)** LFA strip and **(B)** agarose gel electrophoresis, respectively. “+” represents positive; “-” represents negative.

Under the optimized detection condition, the LFA strip was used to evaluate the sensitivity of LFA strip for nucleic acid detection of *Av. paragallinarum*, the bacteria of *Av. paragallinarum* was diluted from 10^4^ to 10^0^ CFU/ml with sterile water before testing. Thereafter, the diluted samples were used for testing by LFA strip and agarose gel electrophoresis, respectively. As displayed in Figure 4A, detection results showed that visible red color on the test line and the control line could be obviously observed in *Av. paragallinarum* sample that were diluted up to 10^2^ CFU/ml, and a slight colorimetric difference could also be shown in the sample diluted up to 10^1^ CFU/ml by using the LFA strip, which could be treated as the visual limit of detection of this LFA strip. Nevertheless, agarose gel electrophoresis showed that it was merely detected when the bacteria concentration was diluted up to 10^3^ CFU/ml and a weak signal was seen in the bacteria sample at 10^2^ CFU/ml (Figure 4B). The results demonstrated that higher sensitivity of LFA strip for nucleic acid detection of *Av. paragallinarum* compared with agarose gel electrophoresis.

### Specificity Analysis of Lateral Flow Assay Strip for Nucleic Acid Detection of *Av. paragallinarum*

**Figure 5:**
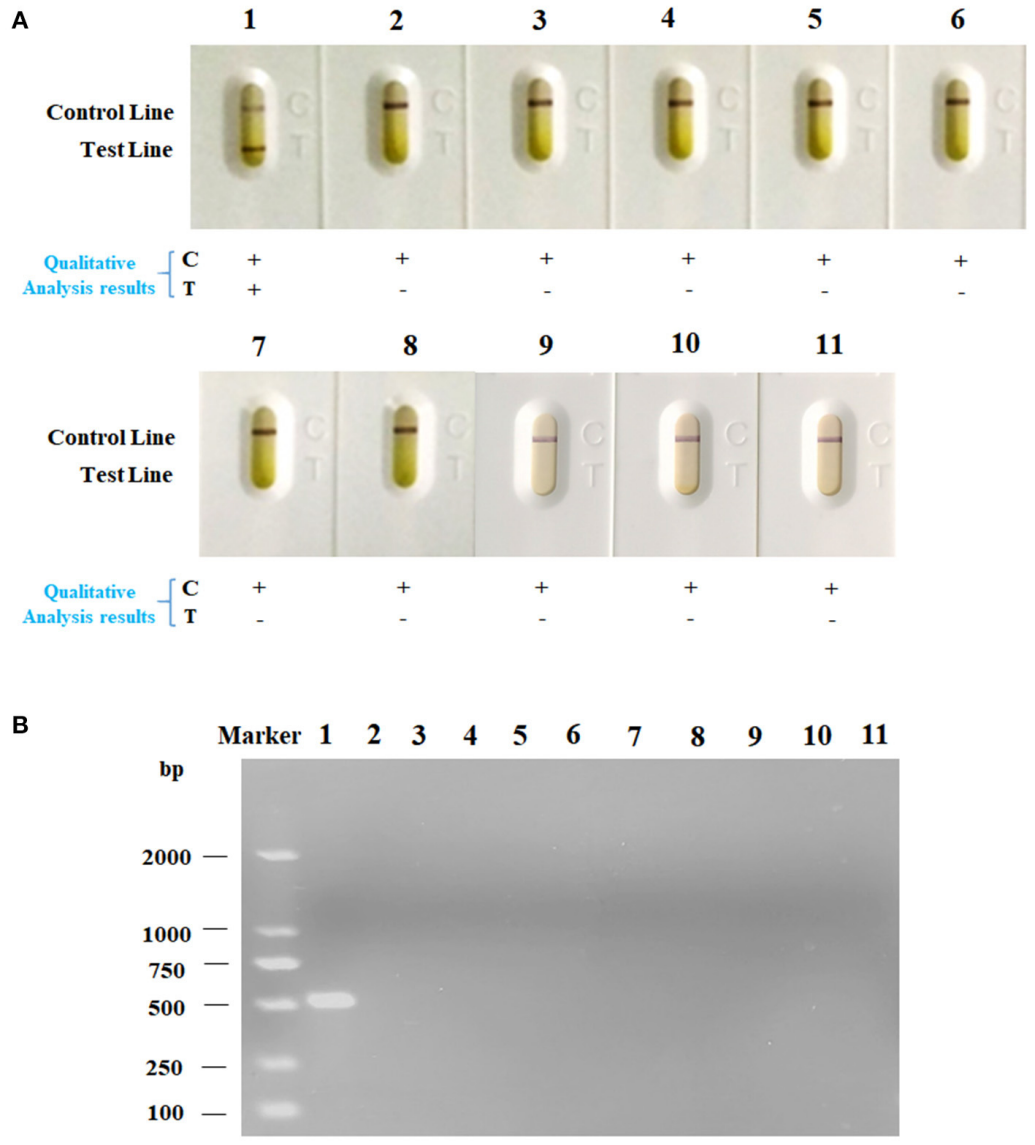
The specificity analysis of LFA strip. The various kinds of bacteria were cultured, and then were detected by **(A)** LFA strip and **(B)** agarose gel electrophoresis, respectively. The names of bacteria samples from 1 to 11 were as follows: *Av. paragallinarum, Ornithobacterium rhinotracheale, Enterococcus faecium, Escherichia coli, Avian streptococcosis, Brodetella bronchiseptica, Pasteurellamultocida, Mycoplasma gallisepticum, Av. gallinarum, Av. Avium*, and *Av. volantium*. “+” represents positive; “-” represents negative.

For the purpose of determining the specificity of the strip, we dropped the strips with various kinds of bacteria that were described above. As shown in Figure 5A, strips dropped with bacteria samples of *Av. paragallinarum* developed a red band on the test line, indicating the positive reaction. However, strips dropped with bacteria samples of *Ornithobacterium rhinotracheale, Enterococcus faecium, Escherichia coli, Avian streptococcosis, Brodetella bronchiseptica, Pasteurellamultocida, Mycoplasma gallisepticum, Av. gallinarum, Av. Avium*, and *Av. volantium* caused no cross reactions that no red band could be displayed on the test line. The specificity of this strip was validated through agarose gel electrophoresis (Figure 5B). The results were consistent with that of agarose gel electrophoresis, indicating the great specificity of LFA strip for nucleic acid detection of *Av. paragallinarum*, which is of great significance for distinguishing between *Av. paragallinarum* and other bacteria.

### Rapid and Accurate Nucleic Acid Detection of *Av. paragallinarum* in Field-Collected Chickens With Infectious Coryza Using Lateral Flow Assay Strip

**Figure 6:**
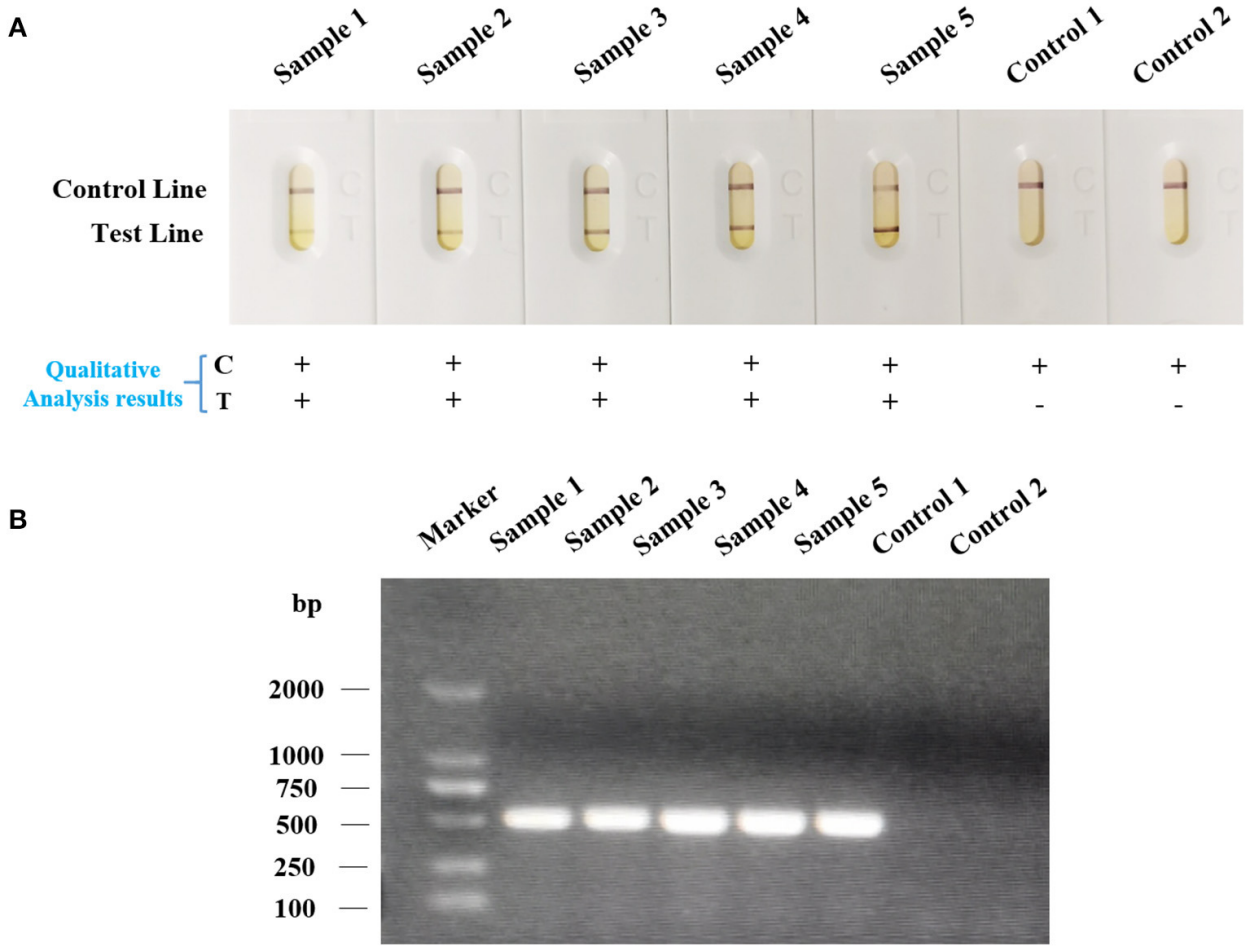
The nucleic acid detection of *Av. paragallinarum* in field-collected chickens suspected of having infectious coryza using LFA strip. The swabs from the infraorbital sinuses of the chickens suspected of having infectious coryza were collected. The samples from healthy chickens were regarded as the control. Subsequently, the bacteria were isolated, cultured, and then were detected by **(A)** LFA strip and **(B)** agarose gel electrophoresis, respectively. “+” represents positive; “-” represents negative.

In order to further confirm whether the LFA strip is capable of being applied for rapid and accurate on-site nucleic acid detection of *Av. paragallinarum*, a total of 60 chicken nasal mucus samples from four different farms were collected as an ideal type of sample for LFA strip assessment (Supplementary Table 1; Figure 6). We found that all the five clinical samples could cause a positive reaction in the LFA strip (Figure 6A). In terms of the samples of healthy chickens, the red band was only present in the control line. The results were also in accordance with that of agarose gel electrophoresis (Figure 6B). Above all, the results confirmed that rapid and accurate on-site nucleic acid detection of *Av. paragallinarum* in clinical chickens with infectious coryza using LFA strip, which can be considered as a reliable tool for diagnosis of the disease of infectious coryza.

### Further Optimization of Lateral Flow Assay Strip Parameters for Better Nucleic Acid Detection of *Av. paragallinarum*

For better nucleic acid detection of *Av. paragallinarum*, strip parameters such as the size and storage condition of nanogold particle, the type, and concentration of immobilized antibodies as well as nanogold-conjugates to compete for, the type and concentration of blocking agent had been optimized in our established LFA strip. However, further efforts remain to be exerted to acquire the best working condition or optimized strip parameters. Additionally, for a good analytical performance, the detection sensitivity, assay accuracy, the strip test reproducibility, and the method robustness should be thoroughly evaluated. In terms of analytical sensitivity, the poor analytical sensitivity also hinders the widespread use of LFA. Previous study has developed a modified LFA assay by integrating sponge shunt into conventional LFA, which is able to make the fluid flow rate decreased and thus achieves the improved analytical sensitivity (35). Maybe it is also suitable for enhancing analytical performance for our established LFA strip with reference to this modification. In our lab, further researches are under way.

## Conclusions

In the current study, we have successfully developed a novel molecular amplification-integrated LFA strip for rapid and accurate determination of nucleic acid of *Av. paragallinarum*. This method perfectly shows the integration of excellent amplification efficiency of PCR and simplicity of LFA strip. The dual dual-labeled double stranded amplicons of the target nucleic acid can be pre-amplified by the use of functional primer set, which can further be visually measured with the LFA strip by the naked eyes. Compared with agarose gel electrophoresis, our established LFA strip is simple to use and time- saving, which displays the visual results within 5–8 min without the need of special instruments and skilled personnel. Besides, this LFA strip has higher sensitivity for nucleic acid detection of *Av. paragallinarum*, which is able to achieve the detection limit of 10^1^ CFU/ml as compared with 10^2^ CFU/ml in agarose gel electrophoresis. In addition, it also has great sensitivity for nucleic acid detection of *Av. paragallinarum* that no cross reaction existed for other bacteria. Furthermore, *Av. paragallinarum* in clinical chickens with infectious coryza were effectively detected by our established LFA strip. Thus, our study is the first to develop a novel LFA strip that can be adopted as a simple, rapid, sensitive, on-site, and specific diagnostic tool to benefit future investigations for the detection of *Av. paragallinarum* and the design of proper prevention and control strategies for this devastating disease, especially in resource-limited areas.

## Data Availability Statement

The original contributions presented in the study are included in the article/Supplementary Material, further inquiries can be directed to the corresponding author/s.

## Ethics Statement

The animal study was reviewed and approved by Animal Care and Use Committee of Animal Husbandry and Veterinary Medicine of the Beijing Academy of Agriculture and Forestry Sciences (IAHVM-BAAFS).

## Author Contributions

CH, DL, ZH, GL, and HS carried out the experiments, analyzed the data, and wrote the paper. CH and HS designed the study, supervised the project, and drew the figures. CH, DL, and HS assisted in the data analysis and discussion. All authors contributed to the article and approved the submitted version.

## Funding

Research was supported by the National Key Research and Development Program of China (Grant no. 2016YFD0500804) and the Beijing Municipal Natural Science Foundation of China (Grant no. 6182010).

## Conflict of Interest

The authors declare that the research was conducted in the absence of any commercial or financial relationships that could be construed as a potential conflict of interest.

## Publisher's Note

All claims expressed in this article are solely those of the authors and do not necessarily represent those of their affiliated organizations, or those of the publisher, the editors and the reviewers. Any product that may be evaluated in this article, or claim that may be made by its manufacturer, is not guaranteed or endorsed by the publisher.

## References

[1] Huo C Zeng X Xu F Li F Li D Li G. The transcriptomic and bioinformatic characterizations of iron acquisition and heme utilization in avibacterium paragallinarum in response to iron-starvation. Front Microbiol. (2021) 12:610196. 10.3389/fmicb.2021.610196 33746913 PMC7970244

[2] Blackall PJ. Infectious coryza: overview of the disease and new diagnostic options. Clin Microbiol Rev. (1999) 12:627–32. 10.1128/CMR.12.4.627 10515906 PMC88928

[3] Page LA. Haemophilus infections in chickens. I. Characteristics of 12 Haemophilus isolates recovered from diseased chickens. Am J Vet Res. (1962) 23:85–95. 14483162

[4] Kume K Sawata A Nakai T Matsumoto M. Serological classification of haemophilus paragallinarum with a hemagglutinin system. J Clin Microbiol. (1983) 17:958–64. 10.1128/jcm.17.6.958-964.1983 6874914 PMC272783

[5] Soriano EV Garduno ML Tellez G Rosas PF Suarez-Guemes F Blackall PJ. Cross-protection study of the nine serovars of haemophilus paragallinarum in the Kume haemagglutinin scheme. Avian Pathol. (2004) 33:506–11. 10.1080/03079450400003502 15545030

[6] Sun H Xie S Li X Xu F Li Y Boucher CE. Selection of avibacterium paragallinarum page serovar B strains for an infectious coryza vaccine. Vet Immunol Immunopathol. (2018) 199:77–80. 10.1016/j.vetimm.2018.04.001 29678233

[7] Zhang PJ Miao M Sun H Gong Y Blackall PJ. Infectious coryza due to haemophilus paragallinarum serovar B in China. Aust Vet J. (2003) 81:96–7. 10.1111/j.1751-0813.2003.tb11445.x 15084021

[8] Xu Y Cheng J Huang X Xu M Feng J Liu C. Characterization of emergent avibacterium paragallinarum strains and the protection conferred by infectious coryza vaccines against them in China. Poult Sci. (2019) 98:6463–71. 10.3382/ps/pez531 31801310 PMC8913951

[9] Kleinman SH Lelie N Busch MP. Infectivity of human immunodeficiency virus-1, hepatitis C virus, and hepatitis B virus and risk of transmission by transfusion. Transfusion. (2009) 49:2454–89. 10.1111/j.1537-2995.2009.02322.x 19682345

[10] Miller DN Bryant JE Madsen EL Ghiorse WC. Evaluation and optimization of DNA extraction and purification procedures for soil and sediment samples. Appl Environ Microbiol. (1999) 65:4715–24. 10.1128/AEM.65.11.4715-4724.1999 10543776 PMC91634

[11] Huber JA Mark Welch DB Morrison HG Huse SM Neal PR Butterfield DA. Microbial population structures in the deep marine biosphere. Science. (2007) 318:97–100. 10.1126/science.1146689 17916733

[12] Chen X Chen Q Zhang P Feng W Blackall PJ. Evaluation of a PCR test for the detection of haemophilus paragallinarum in China. Avian Pathol. (1998) 27:296–300. 10.1080/03079459808419339 18484001

[13] Muhammad TM Sreedevi B. Detection of avibacterium paragallinarum by polymerase chain reaction from outbreaks of infectious coryza of poultry in Andhra Pradesh. Vet World. (2015) 8:103–8. 10.14202/vetworld.2015.103-108 27047005 PMC4777797

[14] Wen S Chen X Xu F Sun H. Validation of reference genes for real-time quantitative PCR (qPCR) analysis of avibacterium paragallinarum. PLoS ONE. (2016) 11:e0167736. 10.1371/journal.pone.0167736 27942007 PMC5152862

[15] Yu Q Zhao Q Wang S Zhao S Zhang S Yin Y. Development of a lateral flow aptamer assay strip for facile identification of theranostic exosomes isolated from human lung carcinoma cells. Anal Biochem. (2020) 594:113591. 10.1016/j.ab.2020.113591 31968209

[16] Oliver C. Colloidal gold/streptavidin methods. Meth Mol Biol. (2010) 588:375–80. 10.1007/978-1-59745-324-0_40 20012851

[17] Paek SH Lee SH Cho JH Kim YS. Development of rapid one-step immunochromatographic assay. Methods. (2000) 22:53–60. 10.1006/meth.2000.1036 11020318

[18] Qian S Bau HH. A mathematical model of lateral flow bioreactions applied to sandwich assays. Anal Biochem. (2003) 322:89–98. 10.1016/j.ab.2003.07.011 14705784

[19] Wang Y Deng R Zhang G Li Q Yang J Sun Y. Rapid and sensitive detection of the food allergen glycinin in powdered milk using a lateral flow colloidal gold immunoassay strip test. J Agric Food Chem. (2015) 63:2172–8. 10.1021/jf5052128 25671495

[20] Shi Q Huang J Sun Y Deng R Teng M Li Q. A SERS-based multiple immuno-nanoprobe for ultrasensitive detection of neomycin and quinolone antibiotics via a lateral flow assay. Mikrochim Acta. (2018) 185:84. 10.1007/s00604-017-2556-x 29594367

[21] Ling S Wang R Gu X Wen C Chen L Chen Z. Rapid detection of fumonisin B1 using a colloidal gold immunoassay strip test in corn samples. Toxicon. (2015) 108:210–5. 10.1016/j.toxicon.2015.10.014 26525659

[22] Cui S Tong G. A chromatographic strip test for rapid detection of one lineage of the H5 subtype of highly pathogenic avian influenza. J Vet Diagn Invest. (2008) 20:567–71. 10.1177/104063870802000505 18776087

[23] Nurulfiza I Hair-Bejo M Omar AR Aini I. Immunochromatographic gold-based test strip for rapid detection of infectious bursal disease virus antibodies. J Vet Diagn Invest. (2011) 23:320–4. 10.1177/104063871102300220 21398455

[24] Li Q Wang L Sun Y Liu J Ma F Yang J. Evaluation of an immunochromatographic strip for detection of avian avulavirus 1 (Newcastle disease virus). J Vet Diagn Invest. (2019) 31:475–80. 10.1177/1040638719837320 30973087 PMC6838702

[25] Yu M Bao Y Wang M Zhu H Wang X Xing L. Development and application of a colloidal gold test strip for detection of avian leukosis virus. Appl Microbiol Biotechnol. (2019) 103:427–35. 10.1007/s00253-018-9461-z 30349931

[26] Yu J Lin Y Cao Y Li X Liao D Ye Y. Development and application of a colloidal gold test strip for the rapid detection of the infectious laryngotracheitis virus. Poult Sci. (2020) 99:2407–15. 10.1016/j.psj.2019.11.066 32359575 PMC7597402

[27] He Y Zhang S Zhang X Baloda M Gurung AS Xu H. Ultrasensitive nucleic acid biosensor based on enzyme-gold nanoparticle dual label and lateral flow strip biosensor. Biosens Bioelectron. (2011) 26:2018–24. 10.1016/j.bios.2010.08.079 20875950

[28] Gao Y Deng X Wen W Zhang X Wang S. Ultrasensitive paper based nucleic acid detection realized by three-dimensional DNA-AuNPs network amplification. Biosens Bioelectron. (2017) 92:529–35. 10.1016/j.bios.2016.10.068 27836603

[29] Mirkin CA Letsinger RL Mucic RC Storhoff JJ. A DNA-based method for rationally assembling nanoparticles into macroscopic materials. Nature. (1996) 382:607–9. 10.1038/382607a0 8757129

[30] Rohrman BA Leautaud V Molyneux E Richards-Kortum RR. A lateral flow assay for quantitative detection of amplified HIV-1 RNA. PLoS ONE. (2012) 7:e45611. 10.1371/journal.pone.0045611 23029134 PMC3448666

[31] Nie W Wang J Xu J Yao L Qiao D Xue F. A molecule capturer analysis system for visual determination of avian pathogenic escherichia coli serotype O78 using a lateral flow assay. Mikrochim Acta. (2020) 187:198. 10.1007/s00604-020-4170-6 32130536

[32] Morales Ruiz S Bendezu J Choque Guevara R Montesinos R Requena D Choque Moreau L. Development of a lateral flow test for the rapid detection of avibacterium paragallinarum in chickens suspected of having infectious coryza. BMC Vet Res. (2018) 14:411. 10.1186/s12917-018-1729-0 30567563 PMC6300026

[33] Chen X Miflin JK Zhang P Blackall PJ. Development and application of DNA probes and PCR tests for haemophilus paragallinarum. Avian Dis. (1996) 40:398–407. 10.2307/1592238 8790892

[34] Ovais M Raza A Naz S Islam NU Khalil AT Ali S. Current state and prospects of the phytosynthesized colloidal gold nanoparticles and their applications in cancer theranostics. Appl Microbiol Biotechnol. (2017) 101:3551–65. 10.1007/s00253-017-8250-4 28382454

[35] Tang R Yang H Gong Y Liu Z Li X Wen T. Improved analytical sensitivity of lateral flow assay using sponge for HBV nucleic acid detection. Sci Rep. (2017) 7:1360. 10.1038/s41598-017-01558-x 28465588 PMC5431006

