# Supplementary material for: A Novel Lateral Flow Assay for Rapid and Sensitive Nucleic Acid Detection of Avibacterium paragallinarum
Source: Front Vet Sci. 2021 Oct 11;8:738558. doi: 10.3389/fvets.2021.738558 (PMC8542899; doi:10.3389/fvets.2021.738558)
Supplement: Supplementary file 1 [file Table_1.DOCX]

**Supporting Information**

**A novel lateral flow assay for rapid and sensitive nucleic acid detection of** ***Avibacterium paragallinarum***

Caiyun Huo^1^, Donghai Li^1,2^, Zhenguo Hu^1^, Guiping Li^1^, Yanxin Hu^2^, Huiling Sun^1*^

^1^ Beijing Key Laboratory for Prevention and Control of Infectious Diseases in Livestock and Poultry, Institute of Animal Husbandry and Veterinary Medicine, Beijing Academy of Agriculture and Forestry Sciences, Beijing, China.

^2^ Key Laboratory of Animal Epidemiology of Ministry of Agriculture, College of Veterinary Medicine, China Agricultural University, Beijing, China.

***** **Corresponding author:**

Dr. Huiling Sun, Beijing Key Laboratory for Prevention and Control of Infectious Diseases in Livestock and Poultry, Institute of Animal Husbandry and Veterinary Medicine, Beijing Academy of Agriculture and Forestry Science, #9 Shuguang Huayuan Zhonglu, Haidian District, Beijing, 100097, China. Email: sunhuiling01@163.com

**Table S1. Comparative description of *Av. paragallinarum* detection in field-collected chickens from 4 different farms by using LFA strip and agarose gel electrophoresis**

| Farm | Chicken | LFA strip | agarose gel electrophoresis |  |
| --- | --- | --- | --- | --- |
| Suspected farm 1 | A1 | **─** | **─** |  |
|  | A2 | **─** | **─** |  |
|  | A3 | **+** | **+** |  |
|  | A4 | **+** | **+** |  |
|  | A5 | **─** | **─** |  |
|  | A6 | **─** | **─** |  |
|  | A7 | **─** | **─** |  |
|  | A8 | **─** | **─** |  |
|  | A9 | **─** | **─** |  |
|  | A10 | **─** | **─** |  |
|  | A11 | **─** | **─** |  |
|  | A12 | **─** | **─** |  |
|  | A13 | **─** | **─** |  |
|  | A14 | **─** | **─** |  |
|  | A15 | **─** | **─** |  |
| Suspected farm 2 | B1 | **─** | **─** |  |
|  | B2 | **─** | **─** |  |
|  | B3 | **─** | **─** |  |
|  | B4 | **─** | **─** |  |
|  | B5 | **─** | **─** |  |
|  | B6 | **─** | **─** |  |
|  | B7 | **+** | **+** |  |
|  | B8 | **─** | **─** |  |
|  | B9 | **─** | **─** |  |
|  | B10 | **─** | **─** |  |
|  | B11 | **─** | **─** |  |
|  | B12 | **─** | **─** |  |
|  | B13 | **─** | **─** |  |
|  | B14 | **─** | **─** |  |
|  | B15 | **─** | **─** |  |
| Suspected farm 3 | C1 | **+** | **+** |  |
|  | C2 | **+** | **+** |  |
|  | C3 | **─** | **─** |  |
|  | C4 | **─** | **─** |  |
|  | C5 | **─** | **─** |  |
|  | C6 | **─** | **─** |  |
|  | C7 | **─** | **─** |  |
|  | C8 | **─** | **─** |  |
|  | C9 | **─** | **─** |  |
|  | C10 | **─** | **─** |  |
|  | C11 | **─** | **─** |  |
|  | C12 | **─** | **─** |  |
|  | C13 | **─** | **─** |  |
|  | C14 | **─** | **─** |  |
|  | C15 | **─** | **─** |  |
| Suspected farm 4 | D1 | **─** | **─** |  |
|  | D2 | **─** | **─** |  |
|  | D3 | **─** | **─** |  |
|  | D4 | **─** | **─** |  |
|  | D5 | **─** | **─** |  |
|  | D6 | **─** | **─** |  |
|  | D7 | **─** | **─** |  |
|  | D8 | **─** | **─** |  |
|  | D9 | **─** | **─** |  |
|  | D10 | **─** | **─** |  |
|  | D11 | **─** | **─** |  |
|  | D12 | **─** | **─** |  |
|  | D13 | **─** | **─** |  |
|  | D14 | **─** | **─** |  |
|  | D15 | **─** | **─** |  |

+: positive results; **─**: negative results
